# Veterinary Medical Association of Oneida County

**Published:** 1894-03

**Authors:** Frank Morrow

**Affiliations:** Secretary; Oneida, N. Y.


### VETERINARY MEDICAL ASSOCIATION OF ONEIDA COUNTY.

A meeting to form a Veterinary Medical Association of Oneida County was held at Stanwix Hall, Rome, Oneida County, New York, on February 14th, 1894. The following veterinarians were present: Drs. Alexander Finley of Camden, R. C. Hurlbut of Boonville, L. G. Moore of Trenton, W. G. Hollingworth of Utica, F. Morrow of Oneida, and Huff and Currie of Rome. The gentlemen met at the invitation of Dr. Huff, who is County Secretary of the New York State Veterinary Medical Society.

The objects of the organization are to hold regular meetings to discuss professional subjects for mutual improvement, and to improve the status of the veterinary profession by bringing its members into more intimate relationship, and consolidating them as a distinctive professional body in the community. Also to regulate veterinary affairs and methods in the education and practice of veterinary medicine, to be in harmony with and conformity to the regulations of the New York State Veterinary Society, and the United States Veterinary Medical Association.

The meeting was called to order at 3:30 o'clock by Dr. Huff, who stated the purposes of the organization It was decided to form an association, and the first order of business was the election of a president Dr. W. G. Hollingworth of Utica was chosen to fill this position. Upon taking the chair he thanked the gentlemen for the honor conferred, and stated some of the benefits which would result from a live organization. He said his idea of the meetings would be to get together to discuss cases and hear papers on different subjects. He remarked that this was the first society to be organized not only in Oneida County, but in this part of the State. He suggested that minutes be kept of different cases, and these minutes could be brought to the meetings and talked over. This would be a great benefit to all.

Dr. Morrow at this point asked if it would be proper for him to join this society, he being a resident of Madison County. Dr. Huff stated that he had been authorized to invite veterinary surgeons of adjoining counties where there is no association at present, and upon that ground he was eligible for membership.

This question being decided the election of a secretary followed. Dr. F. Morrow was elected to fill this office.

The best place for holding meetings was discussed, and it was decided that Rome was centrally located, and inasmuch as Dr. Huff, who has been very active in forming the organization, is a resident of this city, it was thought proper to hold the meetings in Rome. When put to a vote it was so decided.

The time for holding meetings was taken up, and it was concluded to have meetings once every two months, at a date to be fixed by the Secretary.

The question of dues was taken up. It was voted to fix the annual dues at $1 per year, to be increased if necessary. The Secretary was ordered to act as Treasurer.

A communication from Dr. Huidekoper, President of the State Society, was received and placed on file.

Moved and seconded that the by-laws of the society be in harmony with those of the New York State Society. Carried.

Moved and seconded that the Secretary equip himself with the necessary stationery, etc.

A motion was also put and carried that the President read a paper at the next meeting. Members will be expected to make reports of cases.

Dr. Hollingworth read a paper on the subject of the veterinary profession. He told of the advantages of a college course, and stated some of the requisites of a successful veterinarian. Some remarks were made upon professional etiquette. Dr. Hollingworth showed the great advancement made in the profession, and in this connection said all veterinarians should be members of boards of health. He said the only way to rid the country of tuberculosis is to pass the bill making an appropriation, which is now before the legislature. All cattle at fairs should be tested by tuberculine, and local boards of health should also be supplied with it. The veterinary surgeon should be called upon to examine the milk used for sick children. Other contagious diseases were spoken of, and then the doctor spoke a few words about hydrophobia. He said it was purely a contagious disease, and he had met with but one genuine case in his practice, yet we often read of dogs being killed which are supposed to be rabid. Every effort should be made to stop this.

At the conclusion of Dr. Hollingworth's remarks there was some informal discussion of various points, and the meeting adjourned.

FRANK MORROW, Secretary.

ONEIDA, N. Y.

---

